# Assessment of the Effect on Periodontitis of Antibiotic Therapy and Bacterial Lysate Treatment

**DOI:** 10.3390/ijms25105432

**Published:** 2024-05-16

**Authors:** Diana Larisa Ancuţa, Diana Mihaela Alexandru, Florin Muselin, Romeo Teodor Cristina, Cristin Coman

**Affiliations:** 1 Cantacuzino National Medical Military Institute for Research and Development, 050096 Bucharest, Romania; diana.larisa.ancuta@gmail.com (D.L.A.); comancristin@yahoo.com (C.C.); 2 Faculty of Veterinary Medicine, University of Agronomic Sciences and Veterinary Medicine, 050097 Bucharest, Romania; 3 Faculty of Veterinary Medicine, University of Life Sciences “King Mihai I” from Timisoara, 300645 Timisoara, Romania; rtcristina@yahoo.com; 4 Center of Excellence in Translational Medicine, Fundeni Clinical Institute, 022328 Bucharest, Romania

**Keywords:** periodontitis, bacterial lysate, antibiotic therapy, oral microbiome, rat

## Abstract

Periodontitis is an inflammatory process that starts with soft tissue inflammation caused by the intervention of oral bacteria. By modulating local immunity, it is possible to supplement or replace current therapeutic methods. The aim of this study was to compare the effects of an immunostimulatory treatment with the antibiotherapy usually applied to periodontitis patients. On a model of periodontitis induced in 30 rats (divided into three equal groups) with bacterial strains selected from the human oral microbiome (*Aggregatibacter actinomycetemcomitans*, *Fusobacterium nucleatum* and *Streptococcus oralis*), we administered antibiotics, bacterial lysates and saline for 10 days. Clinically, no significant lesions were observed between the groups, but hematologically, we detected a decrease in lymphocyte and neutrophil counts in both the antibiotic and lysate-treated groups. Immunologically, IL-6 remained elevated compared to the saline group, denoting the body’s effort to compensate for bone loss due to bacterial action. Histopathologically, the results show more pronounced oral tissue regeneration in the antibiotic group and a reduced inflammatory reaction in the lysate group. We can conclude that the proposed bacterial lysate has similar effects to antibiotic therapy and can be considered an option in treating periodontitis, thus eliminating the unnecessary use of antibiotics.

## 1. Introduction

Periodontitis is an immune-mediated inflammatory disorder, which can affect up to 45% of the population [1]; globally, it is considered the most common cause of tooth loss [2]. Periodontitis, also called periodontal disease (PD), is caused by biofilms developed subgingivally, destroying oral soft tissue, followed by the progression of infection to the bone, where resorption occurs, resulting in tooth loss [3]. The continuous action of oral microbial agents causes uncontrolled bone metabolism, leading to the release of proinflammatory cytokines, growth factors and cellular messengers [4].

The etiological factors of periodontitis are multiple, similar to periimplantitis, and the common thread is the interaction between bacterial biofilm and the host protector immune response. Risk factors deregulate the microbiome at this level [5], resulting in gingival inflammation and dental biofilm formation [6]. In dental plaque, we find bacteria such as *Streptococcus* spp., which, through expressed adhesins, provide support for the colonization of other bacterial species. Moreover, among the main bacterial agents that trigger periodontitis are *Porphyromonas gingivalis* (P.g), *Tannerella forsythia* (T.f), *Treponema denticola* (T.d), *Aggregatibacter actinomycetemcomitans* (A.a) and *Fusobacterium nucleatum* (F.n) [7,8].

Animal experiments in periodontology are still necessary, and the obtained information is of solid scientific relevance. Animal models are superior to in vitro tests or clinical trials when addressing questions in the area of mechanisms of action and serve as a bridge between hypotheses and the real clinical situation. There is no single animal model that mimics all the characteristics of human periodontitis and its tissue architecture or healing. However, human studies do not allow for the complete evaluation associated with tissue harvesting necessary for microscopic analyses, the importance of which lies in defining the biological impact of regenerative methods and materials. Therefore, research on animal models is necessary to establish cause–effect relationships and, equally important, to test new regenerative devices and advanced therapies [9]. In rats, periodontal disease is an infectious process caused by periodontal pathogens resulting in specific lesions [10]. Thus, rodents are suitable for research targeting teeth or investigating the dynamics of oral inflammatory diseases, and the information gained in this way allows for the translation of basic science into applied research.

In rats, there are several methods to induce periodontitis, starting from tying a cotton or silk thread around the teeth (the most commonly used method), applying acute trauma to the gums or even accelerated induction models that involve placing ligatures contaminated with dental plaque from PD patients [11]. The ligature alone causes, in a very short time, loss of periodontal tissue, biofilm accumulation, ulceration in the sulcular epithelium and eventually infiltration of surrounding tissues. Even if PD is induced by the ligation method without bacterial involvement, bone loss is still dependent on the presence of microorganisms [12]. As a result, in periodontal studies, when rodents are chosen, oral infection with selected human pathogens is used in an attempt to document the virulence of these species [11]. The bacterial composition of periodontitis-associated biofilms comprises a wide diversity of species, an important role of which is played by Streptococcus species that initiate the biofilm and form the basis to which the late colonizers of the red complex (P.g, T.d and T.f) subsequently attach [13].

The development of periodontitis depends on several etiological factors or risk factors, but the relationship between the imbalance of the oral microbiome and the consequent inflammation remains essential in the progression of the disease [14]. The microorganisms responsible for inducing PD trigger the immediate immune response to remedy the dysbiosis and regenerate the damaged periodontal tissue [15]. Thus, the leukocyte lineage is the main pawn controlling the pathogenicity of periodontitis generated by bacterial imbalances, activation of the adaptive immune response and inflammation of surrounding tissues [16].

Treatment of periodontitis, with or without antimicrobial administration, complemented by mechanical plaque removal, is today the gold standard [17]. In early forms of PD, non-surgical therapy and antibiotics may be sufficient, but severe forms require a surgical approach to reduce the periodontal pocket [18]. However, if the focus is only on the involvement of bacteria in the development of PD, current treatments may not always be successful; therefore, current therapies need to be complemented by therapeutic strategies that target the re-enhancement of the immune response capable of remedying the inflammatory and osteolytic process [19].

Drugs targeting the immune system have shown favorable effects in periodontitis [20,21]. Therefore, this study aimed to test a new therapy for periodontitis based on bacterial lysates obtained from strains of A.a, *Streptococcus oralis* (S.o) and F.n, thus evaluating in vivo a new antibacterial treatment for periodontal disease. The experimental model of periodontal disease was created by oral contamination of rats with the same bacteria from which the experimental treatment was obtained.

Null hypothesis: all animals with periodontitis included in this study will heal as a result of bacterial lysate treatment, similar to the effects of antibiotic therapy.

## 2. Results

**Figure 1:**
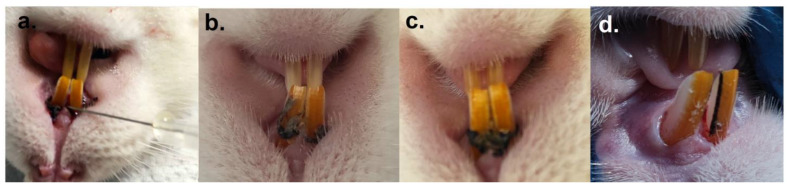
Appearance of periodontal tissue during periodontitis induction period: (**a**). insertion of the ligature wire and its impregnation with bacterial suspension, (**b**). appearance of the gingiva (grayish pink, friable) at 2 weeks after the onset of oral contamination, (**c**). ligature wire with attached dental plaque, observed at 4 weeks of oral contamination, (**d**). appearance of the periodontal pocket at the end of 6 weeks of oral contamination.

The results regarding the induction of PD were similar to those obtained in a previous study [4], the evaluation methods being based on the microbiological examination, especially the clinical one, where we followed the gingival bleeding, the color of the periodontal tissue, the measurement of the periodontal pocket (using periodontal probes model CP 15, KOHLER Medizintechnik Gmbh, Stockach, Germany) and also the observation of any sign of discomfort in mastication expressed by reduced feed consumption and implicitly the reduction in body weight (unpublished data for this study). However, we closely followed the specific signs of periodontitis and as can be seen in Figure 1, compared to the first day, when we fixed the ligatures and started the oral contamination (a), at 2 weeks (b), the appearance of the gums had changed both in terms of color and structure; it became grayish pink, crumbly. At 4 weeks (c), the ligature wire was heavily impregnated with plaque, and bleeding appeared upon probing the periodontal gingival tissue. At the end of the 6 weeks of oral contamination with the selected bacteria, we can observe a visible periodontal pocket (d). All these elements in conjunction with the results of the study we performed previously [4] were sufficient to consider the disease as installed and thus the model to be suitable for treatment testing.

**Figure 2:**
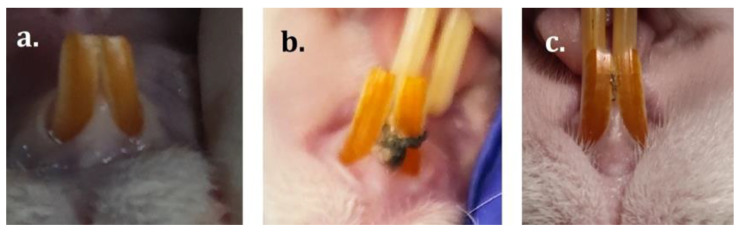
The appearance of the gingival mucosa at the end of the study—(**a**). control group, (**b**). antibiotic and anti-inflammatory group, (**c**). group with bacterial lysate.

During the treatment, the animals showed no signs of discomfort. They were responsive to the doses of the substance established for each group, depending on the weight of the animals (in the case of the antibiotic and anti-inflammatory treated groups) or on the results obtained from the in vitro evaluation when the dose of bacterial lysate was established (in the case of the bacterial lysate-treated group), and no adverse effects were observed. Body weight did not change significantly, and no lesions of particular significance were identified locally. Upon macroscopic examination, the gingiva was similar in color or consistency, regardless of the group (Figure 2). However, bleeding was observed in moderate amounts (similar in antibiotic-treated and lysate-treated groups) when pressure was applied to the periodontal soft tissue.

### 2.1. Hematological Examination Results

We compared antibiotic therapy and bacterial lysate therapy in periodontitis by analyzing neutrophil, lymphocyte, WBC, platelet, hemoglobin, MCH, MCHC and RBC levels. Neutrophils were decreased in both treated groups, in the group receiving bacterial lysate, with a decrease (*p* < 0.001) in circulating blood neutrophil concentration compared to the control group. Lymphocytes decreased irrespective of the therapy applied but without statistically significant relevance. After the application of the treatment, a clear decrease in WBC, RBC and MCHC, moderate reductions in HGB and MCH concentrations and a significant increase in blood platelets could be observed, especially in the bacterial lysate-treated group (Table 1).

**Figure 3:**
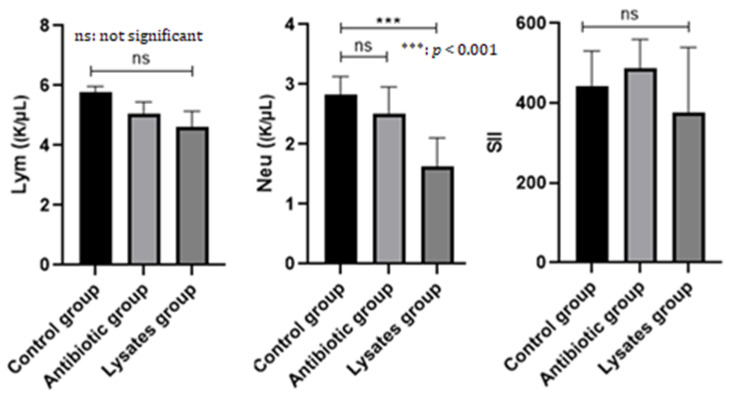
Neutrophils, lymphocytes and SII at the end of the study.

However, by calculating the SII, we observed the maintenance of the inflammatory process even though the statistical analysis did not provide results of significance. The graphically represented SII was higher in the group of animals treated with antibiotic and anti-inflammatory treatment, and by comparison with the group of animals that received bacterial lysate, we can say that this therapeutic approach also provides a considerable reduction in inflammation in the oral tissues (Figure 3).

### 2.2. Immunological Examination Results

**Figure 4:**
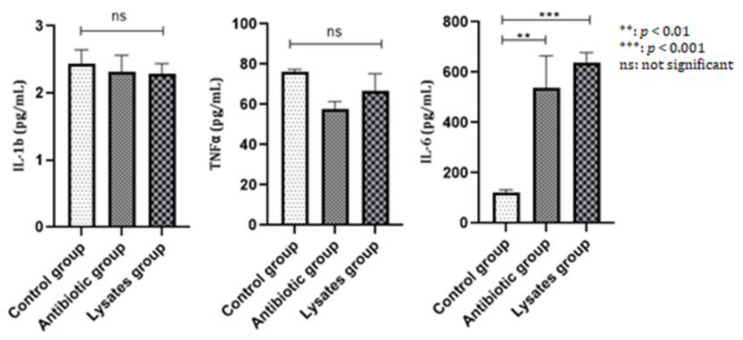
IL-1b, TNFα, and IL-6 at the end of the study.

The immunological examination consisted of an analysis of IL-1, IL-6 and TNFα levels. Related to IL-6, several studies have focused on the potential role of this cytokine in directing the destructive processes in periodontal disease. Reported results have suggested a proinflammatory role for IL-6 alongside IL-1 and TNFα; thus, the biological function of IL-6 related to tissue destruction at the periodontal site differs from that of IL-1 and TNFα and may, in part, play a protective role [22]. This hypothesis applies to our research, where IL-6 levels remained increased (*p* < 0.01) in the antibiotic-treated group, but especially in the group that received bacterial lysate, where *p* < 0.001. The results indicate the organism’s response to the treatment received and indicate similar efficacy for the two therapies. The expressed values of TNFα were close to those of IL-1b, as both the antibiotic and the bacterial lysate caused decreases in TNFα, but without statistical significance (Figure 4).

### 2.3. Microbiological Examination Results

After the oral decontamination step, we found that the bacterial flora was not completely eliminated, which was expected, the residual microorganisms being staphylococcus species (*Staphylococcus xylosus*, *Staphylococcus sciuri*) and micrococci (*Micrococcus luteus*). We thus created a bacterial panel at the onset of the introduction of A.a, F.n and S.o into the mouths of rats. At the end of the 6 weeks of contamination with the selected strains, our concern was to verify the presence of bacteria in the oral cavity.

We selected the strains commonly identified with MaldiTof from the various results. In the control group, it was possible to recover A.a and S.o, but the confidence interval was low, unlike the antibiotic and lysate groups where the oral flora was dominated by commensal bacteria, resistant to the therapeutic means approach, the same as the one we identified after antibiotic therapy with kanamycin and ampicillin (Table 2).

### 2.4. Histopathological Examination Results

Histological sections were blindly evaluated by a histopathologist, and after generating the results, we conducted a semi-quantitative histological assessment to ascertain the host inflammatory reaction, depending on the treatment of each group. The used scoring scheme was adapted from a lesional grade grading system described in mice [23]. The grading methodology was adjusted to account for the host immune reaction observed in rat periodontal tissues, and the total score for each group was calculated by summing the individual scores (Table 3).

**Figure 5:**
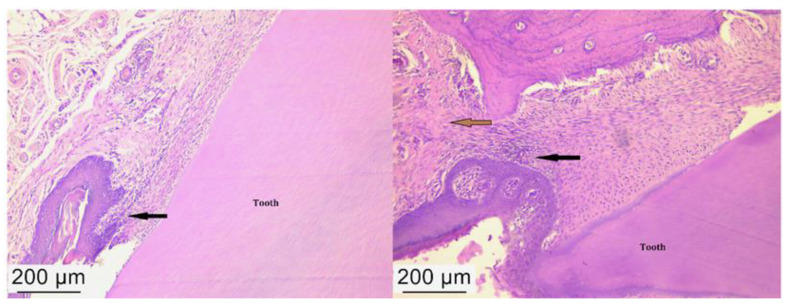
Histological appearance of the periodontal tissues in the control group where inflammatory infiltration can be observed in the periodontal ligament (black arrow) and fibrous tissue (brown arrow). Hematoxylin–eosin staining, ob. 10×.

**Figure 6:**
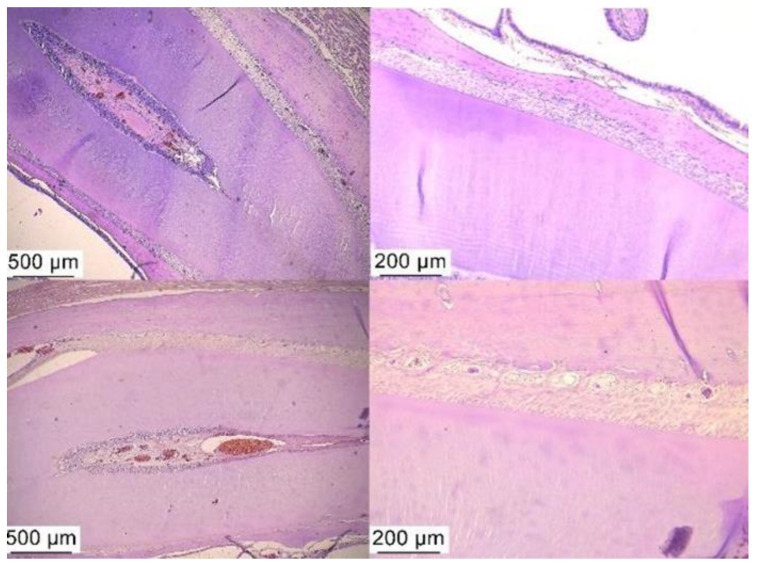
Normal-appearing periodontal tissues were found in groups of animals with antibiotic and anti-inflammatory-treated PD. Hematoxylin–eosin staining, ob. 4× and 10×.

**Figure 7:**
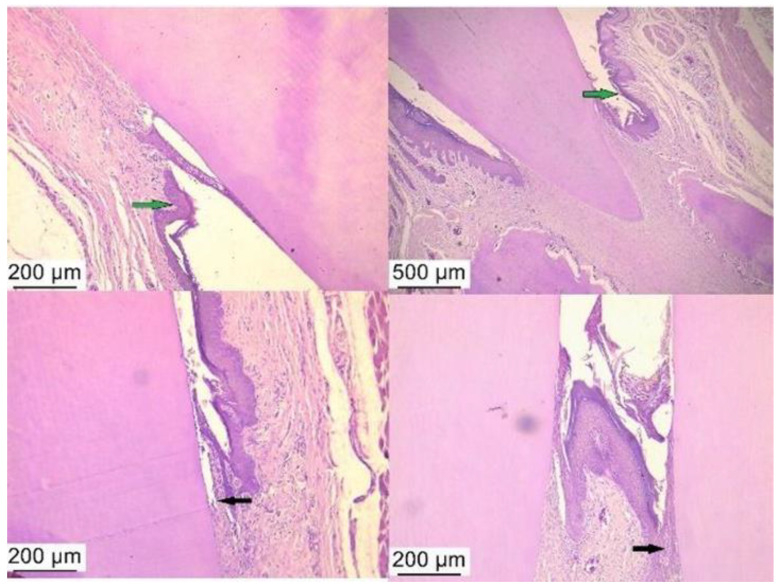
Histological aspect of periodontal tests showing reduced inflammatory infiltrate (black arrow) and periodontal sac free of cellular detritus (green arrow)—group with PD treated with bacterial lysate. Hematoxylin–eosin staining, ob. 4× and 10×.

The control group revealed the junctional epithelium impregnated with neutrophils but also with a rich fibrous tissue (Figure 5). In the antibiotic and anti-inflammatory treated group, we observed an advanced tissue regeneration process, with minimal inflammatory processes (Figure 6) and minimal inflammation in the periodontal tissue after treatment with bacterial lysates (Figure 7).

## 3. Discussion

The use of rats in experimental research brings many benefits, including in the dental field, as the anatomy and physiology of the oral cavity in these animals are similar to those of humans.

In this study, microbiological diagnosis was suggested as a potential method for identifying the most virulent periodontal pathogens [24]. The bacterial species identified in periodontitis are predominantly anaerobic and Gram-negative without being related to a uniform microbial profile [25]. In a healthy organism, the oral bacterial community is in a balanced state with the host, but the intervention of predisposing factors causes bacterial film development and the implicit occurrence of inflammation. Consequently, the increase in the number of strains adhering to the initial biofilm makes therapeutic management difficult [26]. Thus, periodontitis treatment aims to control infection and reduce bacterial substrate. Currently, the standard therapy in PD is the use of systemic antibiotics, which is why we believe that it is necessary to research and develop new therapies to replace antimicrobials, especially in the context of antibiotic resistance, representing an increasingly pressing global problem. Adjuvant therapy for periodontitis is a safe therapy that could also reduce antibiotic consumption. Many studies [27,28] have shown that the use of probiotics shows favorable results, especially when administered preventively, with probiotic strains having the ability to adhere to the periodontium and counteract the activity of oral pathogenic microorganisms. This approach reduces the inflammatory process in the short term [29], which requires more frequent administration [30]. On the other hand, to improve the action of probiotics, knowledge of the individual oral microbiome is necessary so that personalized treatments can be developed [31]. Treatment with antibacterial substances providing immunity against the bacteria responsible for inducing periodontitis should be in the form of vaccines targeting the main virulence factors of microorganisms; so far, vaccination against PD has only been reported in mice [32]. Therapies such as photodynamic, gene and mesenchymal stem cell therapies can slow the progression of periodontal disease by their action on the immune system [33,34,35]. Bacterial lysates act to non-specifically increase the body’s systemic immunity by acting on non-specific defense mechanisms through increased type A antibodies in mucous membranes, phagocytic activity and INF-ƴ production. They may also stimulate the production of specific antibodies against bacterial antigens that compose the bacterial lysate [36]. Based on this consideration, we considered that bacterial lysate therapy can reduce the oral inflammatory process specific to periodontitis.

In this experimental study, clinically, no differences were observed between the antibiotic-treated and bacterial lysate-treated groups. Locally, the gingiva showed slight signs of inflammation, both in the antibiotic-treated and in the bacterial lysate-treated groups, mainly expressed by moderate bleeding upon palpation. Hematologically, we found a decrease in white blood cells in the two treated groups, more pronounced in the animals treated with bacterial lysate. Some observational studies have shown that chronic or aggressive periodontitis is associated with increased white cell counts (mainly neutrophils) and a decrease in red cell counts, and non-surgical periodontal treatments have been associated with decreased white cell counts [37,38]. The results of the present study are consistent with other research which reports that bacterial lysates act similarly to antibiotics to reduce inflammation in the oral cavity [39]. These data reflect the body’s inflammatory response to a periodontium-localized infection, which supports the involvement of periodontitis as a promoter of inflammation. A multitude of mechanisms could explain the association between periodontitis and the increase in leukocytes (neutrophils); more specifically, the inflammatory response of gingival tissue characterized by a leukocyte-dominated infiltrate that can pass into the systemic circulation [40]. Another mechanism addresses the host interaction with pathogenic bacterial flora that determines the chronic production of white matter in the bone marrow [41]. Furthermore, oral bacteria can graft onto existing periodontal lesions, triggering a systemic response to eliminate pathogens. Thus, chemotaxis or phagocytosis are mechanisms that support the destruction of periodontal tissues [42]. The main result of PD is tooth loss, as this disease is characterized by local inflammation that can generate a distant inflammatory process [43]. SII is a relatively new, stable and reliable biomarker that encompasses both localized and systemic immune responses [44]. SII, in recent studies, has been linked to an increased risk of periodontitis [45,46,47], although it was initially correlated with tumor diseases [48]. Chronic inflammatory diseases such as PD, in the case of our study, express a lower SII following treatment with bacterial lysate, without statistical significance compared to antibiotic treatment which denotes that SII can be considered when specific treatments for PD are followed.

Immunologically, potential immunological markers indicating the onset and progression of periodontitis are matrix metalloproteinase (MMP)-8, IL-1, IL-6, TNFα and PGE 2 [49]. The immunological examination performed in this study paid close attention to the biological function of IL-6 and examined its link to periodontal tissue destruction compared to IL-1 and TNFα. In the literature, there are data supporting the gene expression of IL-1 and IL-6 in gingival tissues and relevant protein levels in gingival crevicular fluid, thus confirming an increase in both markers during inflammation [50]. IL-6 is produced by activated T cells or B cells, macrophages, dendritic cells or endothelial cells and fibroblasts [51]. The role of IL-6 has been demonstrated in several diseases, including periodontitis, where it contributes to the development of chondral inflammation, periodontal ligament damage and destruction of bone support [52]. IL-6 is not only a cytokine that responds in the active phase of PD but it is also a potent modulator from the acute to the chronic phase of the inflammatory process [53]. Persistence of IL-6 at high levels may indicate the presence of periodontal pathogenic microorganisms and osteoclastic activity in the dental alveolus. [54,55], IL-6 being directly involved in the pathogenesis of periodontitis [56]. This cytokine reacts to bacterial infections by directing neutrophils and monocytes to the infectious process. Along with IL-6, other proinflammatory cytokines (IL-1, IL-8 or TNF-α) are also associated with osteoclast generation, leading to the development of periodontitis symptoms and/or associated diseases [57,58]. Bone resorption phenomena are initiated by IL-6-mediated factors [56,58], and in the case of treatments applied to periodontitis patients, cytokine levels decrease [59]. In our case, IL-6, following the application of the treatments, remained elevated in the treated groups, demonstrating a similar efficacy of the two treatments. IL1-b and TNF-alpha in attenuated concentrations along with an increased level of IL-6 indicate, however, the body’s effort to ameliorate bone resorption phenomena, providing a protective role against the progression of PD following the applied treatments.

The microbiological examination also showed the positive effect of bacterial lysate so that, at the final identification of oral microorganisms, the strains of A.a, F.n and S.o were no longer isolated. Even though these three bacterial strains were able to induce periodontitis in rats, following antibiotic and lysate treatments, they were not detected. Taking into account that F.n was also not detected in the control group, and that the titer of A.a. and S.o was low, we could think that the bacterial remanence is limited (which should be investigated in future studies) or that the existence of other microorganisms could cover or inhibit the growth of the selected strains on the liquid media.

The methods that can be used to assess the loss of bone support or, in our case, its regeneration, refer to micro-CT, X-rays, photography, densitometry and histology [60]. The degree of destruction of gingival tissue can be easily detected by photographing tissue stained with methylene blue, but with this method, we can obtain a picture based on the influence of external factors. Micro-CT is most often used for multidimensional bone assessment, and other indicators such as bone volume, bone mineral density and trabecular bone parameters can be analyzed without breaking the bone [61,62]. Histological assessment, although laborious, is an effective means of determining the level of internal substances in gingival tissue [63]. In our study, through histological analysis, the detected lesions confirmed the attenuation of the inflammatory process in the periodontal tissues, similarly for both groups that received treatment.

In periodontal research, rats are frequently used as animal models mainly because they have well-characterized biological mechanisms. Specific periodontitis lesions in these animals occur in a short time frame, so researchers can have the necessary support to study new treatment regimens. Over the years, several therapeutic schemes ideal for the treatment of PD have been tested. This topic remains open, and with the results presented in this study, we want to contribute to the improvement in the therapeutic management of one of the most common conditions encountered among patients. Of course, we agree that our proposed product is a new one for use in oral cavity diseases, but the favorable effects obtained in other diseases, such as recurrent respiratory infections [36] and the results obtained in the experiment (concerning antibiotherapy), which support the null hypothesis, encourage us to further investigate bacterial lysates and their effects on oral diseases. In our study, the periodontitis model developed in rats proved to be optimal for analyzing the effects of applied therapy, with the results providing a perspective on the use of bacterial lysates to develop personalized therapy.

## 4. Materials and Methods

### 4.1. Ethics Statement

This study was conducted at the Baneasa Animal Facility (BAF) within the Bucharest Cantacuzino National Medical-Military Institute for Research and Development (INCDMMC). Approval for this study was granted by the Ethics Committee of the Faculty of Veterinary Medicine Bucharest (no 25/15 June 2022), as well as by the veterinary health authority, following EU Directive 63/2010 concerning the care, use and safeguarding of animals utilized for scientific objectives.

### 4.2. Bacterial Cultures and Their Processing

To develop periodontal disease, we selected three bacterial strains (A.a, S.o and F.n), which can form oral biofilm. These came from the bacterial strain bank of the National Institute for Medical and Military Research and Development “Cantacuzino” (CI). Two were authenticated bacteria (A.a—ATCC 29522 and F.n—ATCC 25586), and the third belonged to the German collection of microorganisms and cell cultures (S. o—DSM 20627). All microorganisms used were checked for authenticity, viability, purity and special characteristics to ensure that the experiment was performed to the highest quality.

Bacteria were processed and a cryotube with 1 mL of A.a serogroup b (ATCC 29522) was revived by inoculation into a tube containing Schadler broth medium, left to incubate for a period of one day at 37 °C under anaerobic conditions (80% Nitrogen, 10% Carbon dioxide, and 10% Hydrogen). The suspension density in a tube of unseeded medium was measured with a densitometer (Densitometer McFarland Biosan DEN-1, Riga, Lithuania). The difference determined the A.a strain concentration, amounting to 10^9^ CFU/mL. The rejuvenated A.a culture was preserved in cryotubes at −80 °C, from which daily inoculations were performed throughout the oral contamination period. Specifically, one cryotube of A.a from the primary culture was utilized for each inoculation.

F.n (ATCC 25586) and S.o (DSM 20627) inocula were prepared following the same steps as for A.a, utilizing identical culture media, culture conditions and procedures to establish the density of the inoculum.

The final concentration of each tested strain was 10^9^ CFU/mL, determined by the nephelometric method. The dose to be administered to each animal was 0.6 mL, which included 0.2 mL suspension of A.a, F.n and S.o, and the period over which they were administered was 6 weeks, 5 days/week.

### 4.3. Bacterial Lysate

The method of producing the lysates, as well as the methods of verifying efficacy, have already been described in another manuscript [21]. Briefly, we used the same bacterial strains used to induce periodontitis in the same concentration. Thus, 24 h cultures of A.a, F.n and S.o, at a concentration of 10^9^ CFU/mL, were inactivated on a water bath (56 °C, 60 min). After the inactivation control (when we put 1 mL bacterial lysate belonging to each strain in contact with 9 mL liquid medium of Brain Heart Infusion, Thioglycolate, and Sabouraud which we incubated and daily monitored for possible growth of aerobic or anaerobic bacteria and fungi), which lasted 14 days, the inactivated microorganisms were subjected to the process of mechanical breakage of the bacterial wall by ultrasonication at 70 °C for one hour. The effects of the lysates were tested in vitro by cytotoxicity tests (MTT method) and by contacting lysates (in different concentrations) with live bacteria, the results showing that a double dose of lysate inhibited bacterial growth after 24–48 h.

### 4.4. Animal Selection

Induction of periodontitis was performed on 30 adult male Wistar rats, aged 5 months and weighing an average of 400 g at the start of the study, from the CI’s Specific Pathogen Free (SPF) Animal Facility. Throughout the study, rats were housed under conventional conditions, including nesting material, with unlimited access to food (standard diet produced by the CI’s Combined Feed Mill) and water ad libitum. The animals were housed in groups of five per cage, identified by a tag on which data on species, line, age, sex and cage number were specified, according to the CI’s internal procedures. The general health status of all yearlings was constantly monitored during the experiment.

### 4.5. Periodontitis Rat Model Protocol

Rats were administered kanamycin and ampicillin (20 mg/mL each) in drinking water for 5 days to depress the indigenous microbiota. After the treatment period, saliva samples were collected using cotton swabs from the animals’ mouths to assess the effectiveness of decontamination and to identify any flora that remained unaffected by the treatment. Samples were seeded on liquid culture media (Schadler) and incubated for 24 h at 37 °C under anaerobic conditions. Schadler agar plates were grown from this culture and followed the same thermostat conditions. After the 24 h required for the growth of bacterial colonies, their identification was carried out with the MaldiTof apparatus.

For the induction of PD, we opted to apply ligatures on the upper incisors, employing a gingival retraction thread (UltraPack, Ultradent, Bucharest, Romania), as in a preliminary study [4].

### 4.6. Therapeutic Scheme Applied to Rats with Periodontitis

After completion of the 6 weeks of oral contamination with A.a, F.n and S.o, the rats were divided into three groups of 10, according to the treatment received: control group, treated with saline solution; antibiotic group, treated with amoxicillin + clavulanic acid (40 mg/kg) and acetaminophen (50 mg/kg) and lysate group, which received 1.2 mL bacterial lysate consisting of the three bacterial species, in equal volumes. In all groups, treatment was applied by oral gavage for 10 days.

### 4.7. Animal Monitoring and Analysis

The animals were carefully and clinically monitored throughout the study period for local gingival tissue aspirations or periodontal sac formation. Blood samples were taken from the retro orbital sinus for hematological and immunological examination, and after the study, we euthanized the animals via anesthetic suppression, and samples were collected for microbiological and histopathological examination.

The complete hematological examination was performed on the Idexx Procyte 5diff analyzer on venous blood collected from the retro orbital plexus in vacutainers with EDTA (KIMA Vaccutest, Arzergrande, Italy) at the beginning of the experiment, after 3 weeks of oral contamination and at the end of this procedure. Hematological analysis aimed to follow polymorphonuclear cells (PMN), white blood cells (WBCs), hemoglobin (Hb), red blood cells (RBCs), mean corpuscular hemoglobin (MCH), mean hemoglobin concentration (MCHC), platelet count (PLT) as well as the systemic immune–inflammatory index (SII), calculated with the following formula: SII = NEU × PLT/LYM, which is the product of the number of neutrophils (NEU) and the number of blood platelets (PLT) in relation to the number of lymphocytes (LYM). We preferred to examine this index because it is an easy way (using the results of the blood count) to check for inflammation in the system.

Immunological examination was performed by determining the concentrations of cytokines IL-1b, IL-6 and TNF⍺ in plasma samples collected on day 0 and on the final day of the experiment. This was achieved using the Rat Luminex Discovery Assay, a pre-customized 3-cytokine multiplex assay (LXSARM-3; R&D Systems Inc., Minneapolis, MN, USA). All samples were tested in duplicate. The technique followed the manufacturer’s guidelines.

The MaldiTof bacterial strain identification technique was used for microbiological examination. Rats received ampicillin and kanamycin at the start of the experiment for 5 days to decontaminate the oral cavity and to check for residual bacterial strain types; samples were taken and analyzed in the MaldiTof for identification. Also, at the end of the oral contamination period, we checked for the presence of microorganisms taken in the study using the same method. After, the samples collected from the rats, both at the beginning and at the end of the experiment, were seeded on Schadler culture medium (liquid and solid); then, from the 24 h colonies, the identification of the antibiotic-resistant oral flora and that developed during the experiment was made to follow the presence of A.a, F.n and S.o, along with the commensal microorganisms.

For histopathological examination, the samples collected were fixed in 10% formaldehyde and then decalcified in a decalcifying solution (Histo-Decal, Pantigliate, Italy) for 14 days. After the decalcification period, the samples were processed for paraffin embedding by re-sectioning 5 µm serial sections, followed by staining with hematoxylin–eosin for light microscopic analysis (DM 4000B, Leica, Wetzlar, Germany).

### 4.8. Statistical Analysis

The sample size was calculated a priori using the simplified calculation formula proposed by Arifin et al. supplemented by data entry in the software G*Power 3.1 (Düsseldorf, Germany) where an error α = 0.05% and power of 80% were set [64,65]. Finally, it was decided that the number of animals per group should be 10, considering the complexity of the study related to the induction of periodontal disease, the types of treatments tested, the interval of clinical monitoring of the animals as well as the intervention points for the collection of biological samples (hematological examination).

The normal distribution of the data was analyzed using the Shapiro–Wilk test, after which we performed a statistical analysis of the data by applying one-way ANOVA, Bonferroni test with multiple comparisons between the control and antibiotic group, control and bacterial lysate group. The statistical significance level was set at *p* < 0.05, and GraphPad Prism software (Version 9.4.1, GraphPad Prism Software Inc., La Jolla, CA, USA, released on 26 July 2022) was used.

## 5. Conclusions

This experimental study aimed to compare the effects of antibiotic therapy and bacterial lysate-based therapy in periodontitis induced by oral contamination with A.a, F.n and S.o. For this, we conducted more clinical and paraclinical investigations and the results show that bacterial lysates can be considered strong competitors for antibiotics. By corroborating the results, we can conclude that treatment with bacterial lysates had a similar effect to the standard antibiotic-based periodontitis-specific treatment. Thus, in a situation wherein antibiotic resistance is an increasing problem, the use of bacterial lysates as an alternative is a useful solution worldwide. We recommend further research, development and use of bacterial lysate-based therapies to reduce excessive and unnecessary antibiotic consumption.

## Figures and Tables

**Table 1:** Level of blood parameters before and after application of therapeutic protocols.

Hematological Parameter (Mean ± SD)	Control Group		Antibiotic and Anti-Inflammatory Group		Bacterial Lysates Group	
	Day 0	Final Day	Day 0	Final Day	Day 0	Final Day
WBC, K/µL	12.25 ± 2.83	6.72 ± 1.70	14.00 ± 2.85	10.92 ± 0.30	12.34 ± 4.84	4.0 1 ± 2.29
	*p* < 0.001		*p* < 0.05		*p* < 0.001	
HGB, g/dL	16.76 ± 1.31	16.07 ± 1.09	16.34 ± 0.91	15.1 ± 1.57	15.85 ± 0.77	14.05 ± 3.32
	ns (not significant)		ns		ns	
RBC, M/µL	9.45 ± 0.75	9.32 ± 0.45	9.01 ± 0.73	7.91 ± 0.91	8.73 ± 0.51	8.05 ± 1.81
	ns		ns		ns	
MCH, pg	17.75 ± 0.77	17.22 ± 0.92	18.18 ± 0.98	19.1 ± 0.95	18.15 ± 0.21	17.45 ± 0.21
	ns		ns		ns	
MCHC, g/dL	35.65 ± 0.64	34.5 ± 0.60	36.12 ± 0.63	35 ± 0.45	35.9 ± 0.70	34.8 ± 0.42
	ns		ns		ns	
PLT, K/µL	743.83 ± 169.64	964.75 ± 94.10	616.85 ± 69.27	701.66 ± 512.24	530 ± 11.31	918.5 ± 229.80
	*p* < 0.05		ns		*p* < 0.05	

**Table 2:** The main microorganisms identified with MaldiTof at the end of the study.

ID	Microorganisms Identification	Score
Control 1	*Staphylococcus sciuri*	1.93
Control 2	*Micrococcus luteus*	1.76
Control 3	*Aggregatibacter actinomycemcomitans*	0.57
Control 4	*Streptococcus oralis*	1.45
Control 5	*Staphylococcus xylosus*	2.87
Antibiotic and anti-inflammatory group 1	Unidentified organism	1.45
Antibiotic and anti-inflammatory group 2	*Corynebacterium minutissimum*	1.63
Antibiotic and anti-inflammatory group 3	Unidentified organism	0.22
Bacterial lysates group 4	Unidentified organism	1.19
Bacterial lysates group 5	*Staphylococcus epidermidis*	1.84
Highly reliable identification		2.00–3.00
Low confidence identification		1.70–1.99
Not possible to identify organism		0.00–0.69

**Table 3:** Semi-quantitative scoring for histological evaluation in groups of treated animals.

Parameter	Score	Incidence of Lesions	Group
Inflammatory infiltrate in periodontal connective tissue	0	No neutrophils/macrophages in the microscopic field	Antibiotic and anti-inflammatory group Bacterial lysates group
	1	Reduced neutrophil/macrophage	Antibiotic and anti-inflammatory group Bacterial lysates group
	2	Moderated neutrophil/macrophage	-
	3	Neutrophil/macrophage marked	Control group
Fibrosis	0	Absent	-
	1	Mild	-
	2	Moderate	Antibiotic and anti-inflammatory group Bacterial lysates group
	3	Severe	-

## Data Availability

Data are contained within the article.
